# Bovine Trypanosomosis and Its Vectors in Three Selected Districts of Buno Bedele Zone of Oromia Region, Ethiopia

**DOI:** 10.1155/2020/1571947

**Published:** 2020-07-25

**Authors:** Gelaye Gebisa, Kibiru Beriso, Biruk Bogale, Oda Gizaw, Dawit Chala

**Affiliations:** ^1^School of Animal and Range Sciences, Hawassa University, Awassa, Ethiopia; ^2^Department of Animal Science, Mettu University, Mettu, Ethiopia; ^3^National Tsetse and Trypanosomiasis Investigation and Control Centre, Bedele, Ethiopia

## Abstract

Trypanosomosis is one of the most economically challenging diseases affecting mammals, and it is a serious haemoprotozoan disease caused by different species of unicellular eukaryotic parasite of the genus trypanosome. The study was conducted to access the prevalence of bovine trypanosomosis, its associated risk factors, and vector density on cattle reared in three selected districts, namely, Chewaka, Dabo Hana, and Meko districts. Blood was collected from a total of 1046 cattle of age groups extending from 1 to 6 years. The buffy coat technique was used to check the presence of parasites from sampled blood, and the trypanosome species were identified using Giemsa-stained thin blood films. The packed cell volume of sampled blood was determined using the haematocrit. A total of 160 traps were deployed to study the entomological survey. Generally, 3.44% of the studied animal was infected with trypanosomosis, and *T. vivax* was the dominant species of trypanosomosis in the study areas. Significant differences (*P* < 0.05) were observed due to associated factor viz. body condition and anaemic status of the animal; however, insignificant differences were also recorded between different districts, age group, and sex. The mean PCV value of parasitaemic and aparasitaemic animals was 22.22 ± 0.92 and 26.18 ± 0.16, respectively, and significant difference was *P* < 0.05. An overall of 1.82 flies per trap per day was recorded from the study areas, and among the total caught vectors, 81.4% of it was *G. tachinoides* and the rest was *G. morsitans*. Therefore, the veterinarians have to continue providing the appropriate medication/treatment for the infected animals per appropriate recommendation, and Bedele NTTICC has to take more measures to control the density and distribution of tsetse flies in Dabo Hana district than the others due to high flies per trap per day observed in Dabo Hana district.

## 1. Introduction

Among the constraints associated with animal health, trypanosomosis is one of the important health factor results for low or insignificant livestock production in the area of Africa, which has the greatest potential for significant increases in domestic livestock productivity [1]. It is a serious haemoprotozoan disease caused by different species of unicellular eukaryotic parasite of the genus trypanosome found in the blood and other tissues of vertebrates including livestock, wildlife, and people and is transmitted cyclically by tsetse flies of Glossina species and many other insects mechanically [2, 3]. Bovine trypanosomosis is one of the major obstructions to livestock development and agricultural production in Ethiopia as causative for insignificant development in general and to food self-reliance efforts of the nation in particular [1]. It is also a cause for the severe and frequently fatal disease of livestock mainly in the poor rural community, and it is fairly considered as a foot root cause of poverty in Ethiopia.

Vector born trypanosome species are disseminated in most parts of western and southwestern parts of Ethiopia [1, 4]. Tsetse flies are the cause for the transmission of trypanosomosis from one animal to others and widespread in the western, south, and southwestern lowland regions and the associated river systems, i.e., Abay, Gibe Omo, and Baro [5]. Presently, about 220,000 km^2^ areas of the above-listed regions are infested with five species of tsetse flies namely *Glossina pallidipes*, *G. morsitans*, *G. fuscipes*, *G. tachinoides*, and *G. longipennis* [6]. A major determinant of the distribution and epidemiology of bovine trypanosomosis is the availability of suitable habitat for tsetse. Tsetse is restricted to various geographical areas according to habitat and most of them distributed in forest, riverine, and savannah areas. The palpalis and morsitans species are dominantly exist in the major livestock rearing areas and have great importance in veterinary practices [7].

The most prevalent trypanosome species in tsetse infested areas of Ethiopia were *Trypanosoma congolense* and *Trypanosoma vivax* [8]. The reported prevalence varies from locality to locality depending on agroclimatic conditions, seasons, and as part of activities, which were intended to control the impact of the disease [5]. Among all mammals, bovines were dominantly affected by trypanosomosis, and it has more importance in regards to economic point views [7].

The economic loss from crop and livestock production was directly or indirectly affected by trypanosomosis [8]. It is a severe problem for agricultural production in widespread areas of the tsetse infested regions and non-tsetse transmitted trypanosomosis, which affects a considerable number of animal populations in tsetse free zone of the country [9]. Among the total regions of Ethiopia, Amhara, Benishangul Gumuz, Gambella, Oromia, and SNNPR regions are mostly infected with more than one species of tsetse flies [10, 11]. Even if trypanosomosis is a very important disease, not enough studies have been conducted in Buno Bedele zone of Oromia Region. However, within the same zone, there is no study conducted so far in Chewaka, Dabo Hana, and Meko districts. Thus, this study was conducted in the above-listed three selected districts of Buno Bedele zone with the objectives of determining the prevalence of bovine trypanosomosis and the influence of associated risk factors.

## 2. Materials and Methods

Description of the study area: the study was conducted in three selected districts of Buno Bedele zone of Oromia Region, Ethiopia. Geographical locations of the studied locations are presented in Table 1. The dominant crops in the study areas are maize (*Zea mays*), teff (*Eragrostis tef*), coffee (*Coffea arabica*), sorghum (*Sorghum bicolor*), barley (*Hordeum vulgare*), wheat (*Triticum* spp), rice (*Oryza sativa*), different pulse crops, finger millet (*Eleusine coracana*), fruits, and different types of vegetables and spices. Most of the residents in the area are dependent on agrarian activities to a greater or lesser extent [12], and crop and livestock sales are the important sources of income for all wealth groups [13].

Study design: a cross-sectional study was conducted to determine the prevalence of bovine trypanosomosis in selected three districts of Buno Bedele zone in the study period from 4 June 2019 to 5 November 2019. The study constituted the local cattle of different age groups, body condition scores, and both sex groups of cattle from three selected districts of study areas. The age of the cattle was determined according to the defining characteristics [14] and information from owners of the cattle. The body condition of the study animals was categorized based on the criteria described by [15].

Sample size determination and sampling strategies: multistage random and proportional purposive sampling techniques were employed to select the representative animal from the study areas. The random sampling method was employed to select the three districts (Chewaka, Dabo Hana, and Meko) from the whole Buno Bedele zone of Oromia Region. The peasant associations (PA's) involved in the study areas were chosen using a proportional purposive sampling method based on the density of cattle population. Accordingly, from Chewaka district (Waltasis, Jagan, Sire Gudo, Burka Anani, and Dabena), Dabo Hana district (Didessa, Loko, and Lilo), and Meko district (Biftu Nega, Oda Chekorsa, and Kodi Gas) were the involved peasant associations during the study periods. Finally, the studied animals were selected using simple random sampling techniques. The number of animals required for the study was assessed using the formula given by Thrusfield [19] for simple random sampling.

(1) $$\begin{array}{c} N=\frac{1.96^{2}*P_{\exp}\left(1-P_{\exp}\right)}{d^{2}}, \end{array}$$

where *N* = required sample size, *P*_exp_ = expected prevalence, and *d* = desired absolute precision.

The sample size determination was using a 95% level of confidence, 50% expected prevalence, since there was no previous study conducted in the selected three districts (Chewaka, Dabo Hana, and Meko), and 0.05 desired absolute precision. Based on the formula, the sample size would have been 384 cattle; however, 1,046 cattle were involved as a sample for increasing the precision of the study.

### 2.1. Study Methodology and Procedures

#### 2.1.1. Buffy Coat Technique

Blood was collected from an ear vein using a heparinized microhematocrit capillary tube, and the tube was sealed. A heparinized capillary tube containing blood was centrifuged for 5 min at 12,000 rpm. After the centrifugation, trypanosomes were usually found in or just above the buffy coat layer. The capillary tube was cut using a diamond-tipped pen 1 mm below the buffy coat to include the uppermost layers of the red blood cells and 3 mm above to include the plasma. The content of the capillary tube was expressed on to slide, homogenized on to a clean glass slide, and covered with a coverslip. The slide was examined under *a* ×40 objective and ×10 eyepieces for the movement of the parasite [20].

#### 2.1.2. Thin Blood Smear

The trypanosome species were identified using Giemsa-stained thin blood films. A small drop of blood from a microhaematocrit capillary tube to the slide was applied to a clean slide and spread by using another clean slide at an angle of 45°, air-dried, and fixed for 2 min in methyl alcohol, and then immersed in Giemsa stain (1 : 10 solution) for 50 min. Drained and washed off the excess stain using distilled water, allowed to dry by standing upright on the rock, and examined under the microscope with the oil immersion objective lens. This technique is the most sensitive of the parasitological tests for the detection of *T. vivax* and *T. congolense* [21, 22]. However, the microcentrifugation technique [23] is several times more sensitive than thin-stained blood smear for diagnosis of animal trypanosomosis.

#### 2.1.3. Measurement of Packed Cell Volume (PCV)

Blood samples were obtained by puncturing the marginal ear vein with a lancet and collected directly into a capillary tube. The capillary tubes were placed in a microhaematocrit centrifuge with sealed end outermost. The tube was loaded symmetrically to ensure good balance. After screwing the rotary cover and closing the centrifuge lid, the specimens were allowed to centrifuge at 12,000 rpm for 5 min. Tubes were then placed in haematocrit, and the readings were expressed as a percentage of packed red cells to the total volume of whole blood. Animals with PCV ≤24% were considered as anaemic [24].

#### 2.1.4. Entomological Survey

A total of 137 monopyramidal, 11 Ngu, and 12 biconical traps were positioned in twelve peasant associations of the selected three districts. Each of them was placed with an approximate interval of 100–200 m for 48 hrs in watering and grazing points, in which the fly and the vector are believed to have frequent contacts. A mixture of acetone, octanol, and cow urine was used as a bait to attract the flies. Then, after 48 hrs of deployment, tsetse flies in the cages were counted and identified based on their habitat and morphology to the genus and species level [25]. The sex of tsetse flies was identified by observing the posterior end of the ventral aspect of the abdomen using a hand lens. Male flies were identified by their enlarged hypopygium in the posterior ventral end of the abdomen. The apparent density of the tsetse fly was calculated as the number of tsetse catch/trap/day [26].

#### 2.1.5. Data Analysis

The collected data were analyzed using SPSS (version 20 : 0). Descriptive statistics was employed to measure the prevalence of trypanosomosis and existing parasite species in the study areas. The chi-square test was employed to test the significant difference of prevalence of trypanosomosis and mean PCV values in association with factors such as sampling areas (districts), age, body condition, sex, and anaemic status of the studied animals. The independent *t*-test was utilized to compare the mean PCV values of the parasitemic and aparasitemic animals. A single factor ANOVA was employed to test the mean PCV values of an animal infected with different parasite species and noninfected animal. Differences between parameters were tested for significance at probability levels of *P* < 0.05 and 95% confidence interval. The apparent density of the tsetse fly was calculated as the number of tsetse catch/trap/day.

## 3. Result and Discussion

### 3.1. Result

#### 3.1.1. Parasitological Findings

From a total of 1046 cattle, 36 of them were diagnosed as positive for infection using a buffy coat technique with an overall prevalence of 3.44%. Among the overall infected animals, 2.78, 2.82, and 4.49% of them were found in Chewaka, Meko, and Dabo districts. *T. vivax* (52.78%) was the most prevalent trypanosome species, and the rest (47.22%) was recorded as *T. congolense* species (Table 2).

#### 3.1.2. Prevalence in Association to Different Factors

Effect of different associated factors on the prevalence of trypanosomosis is indicated in Table 3. The prevalence of trypanosomosis in sampling areas of Chewaka, Meko, and Dabo Hana districts was 2.78%, 2.82%, and 4.49%, respectively, and it was not significantly different (*P* > 0.05) across the districts. Similarly, there was no significant difference among the different sex and age group of cattle in trypanosomosis infection. Among 1046 sampled animals, 5.47%, 2.84%, and 1.47% prevalence of bovine trypanosomosis was recorded as a poor, medium, and good body condition, respectively. The difference (*P* < 0.05) between poor, medium, and good animals in body condition was statistically significant. Among the total examined animal, 630 and 416 of them were nonanaemic and anaemic cattle, and they were diagnosed as a positive for trypanosomosis with the proportion of 1.75% and 6.01%, respectively. The association between being anaemic and nonanaemic animal significantly influence the prevalence of trypanosomosis at *P* < 0.05.

#### 3.1.3. Hematological Results of Infected and Noninfected Animals

The recorded mean PCV value of aparasitaemic and parasitaemic animals indicated that out of 1,046 examined animals, 36 of them were found as anaemic (parasitaemic) animals, and their mean PCV value was statistically (*P* < 0.05) lower than that of aparasitaemic (noninfected) animals. The observed mean PCV value of animals infected with *T. vivax* and noninfected animal was statistically different at *P* < 0.05, and insignificant differences (*P* > 0.05) were observed between animal infected with *T. congolense* and *T. vivax* as well as noninfected (Table 4).

#### 3.1.4. Entomological Survey

A total of 160 traps were deployed for two consecutive days at 12 peasant association's (PA's) in three selected districts, and a total of 1,751 *Glossina* species of flies were caught. Among the caught flies, 18.6% and 81.4% of them were *G. morsitans* and *G. tachinoides*, respectively. The overall apparent tsetse fly density was 1.82 flies/trap/days. The flies' density per different study sites was 2.73, 0.33, and 11.65 flies/trap/days in Chewaka, Meko, and Dabo Hana districts, respectively. The highest density of flies/trap/days was recorded in the Dabo Hana district and lowest in Meko district. Among the caught flies, 32.9% and 67.1% of the flies were male and female, respectively (Table 5).

## 4. Discussion

This study had revealed the overall prevalence of trypanosomosis in the study areas was 3.44%. The result of this finding was almost in line with Adane and Gezahagne [27] who reported a 3.5% prevalence of trypanosomosis in Dejen district, Amhara Region, Ethiopia. The result of this finding was lower compared to other studies conducted somewhere else within the same country, which varies from 4.43 to 14.97% in a year between 2012 and 2019 [1, 28–33]. The overall low record of the prevalence of trypanosomosis may be due to the impact of parasite, and vector control practices have been working by Bedele NTTICC (Bedele National Tsetse and Trypanosomiasis Investigation and Control Centre) and the geographical location of the study areas, which have equal access for getting an extension service to the farmers regarding how to control the distribution of vectors (tsetse flies), disease control, and treatments used for treating the infected animal. The suggestions are similar with [5, 8] who stated that the prevalence varies from locality to locality depending on activities, which were intended to control the impact of the disease.

The present study in terms of *Trypanosome* species is in agreement with the findings reported by Tewodros [34] who reported the highest proportion of *T. vivax* from Hawa Gelan district, Oromia Region, Ethiopia. According to his report, 45.85% and 33.33% of *T. vivax* and *T. congolense* were recorded, respectively. In contrast to this, the highest proportion of *T. congolense* was recorded on the report of Megersa et al. [33] and Marta et al. [30] who reported a high proportion of *T. congolense* from Botor Tolay and Chora districts of Oromia Region, Ethiopia, respectively.

The significant difference was not observed in the prevalence of trypanosomosis between animals sampled from the study districts during a study period (*P* > 0.05). The finding of this study is in agreement with the study of Zemedkun [35] and Abayneh [1] those who reported from Kindo Koysha district of Wolayita Zone and three selected districts of Wolayita Zone, Ethiopia. In contrast to this, a significant difference among different study site was reported by Abebayehu and Biniam [28] from two districts of Bench Maji Zone, southwestern Ethiopia. The observed insignificant result could be due to alike controlling measures that had been taken to control the tsetse flies densities by Bedele NTTICC, the geographical location of districts being adjacent to each other and having the same flies belt and the same treating strategies. This is similar with the suggestion provided by [5, 8] who stated that the prevalence varies from locality to locality depending on activities, which were intended to control the impact of the disease and way of treating the infected animal.

Among the total (37) infected animals, 69.44 and 30.56% of them were anaemic and nonanaemic cattle, and the difference was statistically significant at *P* < 0.05. The result of this finding is in agreement with the finding of Marta et al. [30] who reported that the highest prevalence of trypanosomosis from anaemic cattle in Chora districts of Oromia Region, Ethiopia. The higher prevalence of infected animal in association to being anaemic might be attributed to the sampled animals which got diseases such as helminthosis, tick-borne diseases, and nutritional imbalances, and it is in accordance to the suggestion provided by [28].

The significant difference was not observed in the prevalence of trypanosomosis between the different age groups of cattle during the study period. The finding of this study is in line with the study reported by [1, 31, 34]. They found an insignificant effect of age on prevalence of trypanosomosis on cattle sample from three selected districts of Wolayita Zone, Kindo Koysha district of Wolayita Zone, and Hawa Gelan district of Oromia Region, Ethiopia, respectively. However, the study reported by [30, 33] indicated that there is a significant difference in the prevalence of trypanosomosis among the different age groups of sampled animals in the study areas. This might be due to similar regulatory measures that had been taken to control the tsetse flies densities by Bedele NTTICC and similar extension services concerning disease control and treating all age groups of infected animals. The suggestion is alike to [5, 8] who stated that the prevalence varies from locality to locality depending on activities, which were intended to control the impact of the disease.

There was no significant difference (*P* > 0.05) between animals of different sex group. The result of this finding is in agreement to the study reported by Eskziaw et al. [31] from Gimbo and Guraferda districts of southern Ethiopia, Megersa et al. [33] from Botor Tolay district of Jimma Zone, Ethiopia, and Marta et al. [30] from Chora district of southwestern Oromia, Ethiopia. However, a significant difference was recorded by Zemedkun et al. [35] from three selected districts of Wolayita Zone, southern Ethiopia. This could be attributed to similar controlling measures that had been taken to control the tsetse flies densities by Bedele NTTICC and alike extension services concerning disease control and treating all sex groups of infected animals. The suggestion is similar to [5, 8] who stated that the prevalence varies from locality to locality depending on activities, which were intended to control the impact of the disease.

The prevalence of trypanosomosis was statistically different (*P* < 0.05) across a different group of body condition. The result of this finding is consistent to the study reported by Zemedkun et al. [35] and Megersa et al. [33] who reported that there was a significant difference in the prevalence of trypanosomosis among cattle categorized according to their body condition from Kindo Koysha district of Wolayita Zone and Botor Tolay district of Jimma Zone, Ethiopia, respectively. In contrast to this, the insignificant effect of body condition on the prevalence of trypanosomosis on cattle from Kindo Koysha district of Wolayita Zone, Ethiopia, is recorded by [1]. These differences might be due to the disease which itself results in progressive emaciation of infected animals [36–38].

Among the total (1046) examined animal, 36 of them were parasitemic, and they were found to be anaemic (PCV ≤ 24%) compared to aparasitemic animals, and the difference was statistically significant (*P* < 0.05). The result of this finding is in accordance to the study reported by Van den Bossche and Rowlands [39] who stated that the average PCV of parasitologically negative animals was significantly higher than that of parasitologically positive animals. Thus, trypanosomosis may be involved in adversely lowering the PCV value of infected animals. The low PCV value of infected animals may be due to the animals that were getting sick with diseases such as helminthosis, tick-borne diseases, and dietary disparities. It is similar to the suggestion provided by [28]. Similarly, the mean PCV value of animal infected with parasites *T. vivax*, and noninfected animals were significantly different (*P* < 0.05). However, insignificant difference (*P* > 0.05) was also recorded between an infected animal with *T. congolense* and the rest noninfected and animal infected with the *T. vivax*. The result of this finding is in agreement with the study report of Zemedkun et al. [35] from three selected districts of Wolayita Zone, southern Ethiopia. This can be due to *T. vivax* attacks other tissues beside to blood such as the lymph node, eyes, and heart, but *T. congolense* limited in the blood that might be resulted for low PCV value compared to the *T. vivax*, and this is in line with the suggestion given on the study report of [35, 37, 40, 41].

The apparent density of tsetse flies was about 1.82 flies/trap/day. Different types of traps were designed to catch different species of tsetse flies. The traps availability is indicated based on their efficiency for those species for which the trap has been tested and recommended [42]. Among the total 1751 (*Glossina* species), 18.6% (*G. morsitans*) and 81.4% (*G. tachinoides*) were caught during two consecutive trapping days. Among a total of 160 traps, 137, 11, and 12 of them were monopyramidal, Ngu, and biconical traps, respectively, and each of them had different efficiency in catching different species of *Glossina* species. The apparent tsetse fly density recorded from Chewaka district (2.73) is close to the report of Abebayehu and Biniam [28] who reported 2.83 from two districts of Bench Maji Zone, western Ethiopia. However, the highest density was observed in Dabo Hana district (11.65 F/T/D), and it is also in agreement to the finding of Megersa et al. [33] who reported (11.6 F/T/D) from Botor Tolay district, Jimma Zone, Ethiopia. The overall finding of this study is lower than the finding of Ayele et al. [43] from Daramallo district, Ethiopia, with flies/trap/day of 19.14 and 14.97 recorded from selected different study sites of Arbaminch by [44]. In contrast to this, the lower (0.14 flies/trap/day) density was recorded by Zemedkun et al. [32] from three selected districts of Wolayita Zone, southern Ethiopia. This difference might be ascribed to somewhat agroecological differences and season in the study area.

## 5. Conclusions

The finding of this study showed that trypanosomosis is one of the challenging diseases affecting the agricultural activity of farmers, and it results in getting low income from cattle rearing in the study areas. *T. vivax* and *T. congolense* were the type of parasite species recorded in the study areas, and the highest proportion was observed as *T. vivax.* This study also indicates that there was a significant association between the prevalence of trypanosomosis and risk factors viz. body condition and anaemic status of the animal. However, age group, sex, and study sites (districts) did not significantly affect the prevalence of trypanosomosis in the study areas. The mean PCV value of infected animal was lower compared to that of the normal animal, and significant differences were recorded. The *Glossina* species, particularly *G. tachinoides* and *G. morsitans* were the type of *Glossina* species existing in the study areas, and the former has dominantly existed in the study areas. Therefore, the veterinarians have to continue providing the appropriate medication/treatment for the infected animals per appropriate recommendation, and Bedele NTTICC has to take more measures to control the density and distribution of tsetse flies in Dabo Hana district than others due to high flies per trap per day observed in Dabo Hana district.

## Figures and Tables

**Table 1:** Geographical information of the studied districts.

Variables	Buno Bedele zone		
	Dabo Hana	Meko	Chewaka
Altitude (m)	1773	2226	900–1400
Highland (%)	10	53.3	11
Midland (%)	66	23.3	22.4
Lowland (%)	24	23.3	66.6
Latitude	N08°67′30″–N08°71′21″	N08°41′25″	N08°54′40″
Longitude	E36°41′89″–E36°40′33″	E36°1′44″	E36°09′18″
Rainfall range/average (mm)	1131	1200	1000–1100
Temperature range/average (°C)	18–24	8–28	24
Distance from Addis Ababa (km)	534	566	552
Sources	[16]	[17]	[18]

**Table 2:** The overall prevalence of trypanosome in the study areas.

Variables	Description	Number of examined	Prevalence in number (%)
Districts	Chewaka district	432	12 (2.78)
	Meko district	213	6 (2.82)
	Dabo district	401	18 (4.49)
	Total	1046	36 (3.44)

Parasite species	*T. vivax*		19 (52.78)
	*T. congolense*		17 (47.22)
	Total		36 (100.00)

**Table 3:** The prevalence of trypanosome and effect of associated risk factors in the study areas.

Factor	Number of animals examined	Prevalence in number (%)	*χ* ^2^	*P* value
Study sites				
Chewaka	432	12 (2.78)	2.146	0.342
Meko	213	6 (2.82)		
Dabo Hana	401	18 (4.49)		

Status				
Nonanaemic	630	11 (1.75)	13.705	0.000^*∗*^
Aneamic	416	25 (6.01)		

Sex				
Male	652	20 (3.53)	0.729	0.393
Female	394	16 (3.30)		

Body condition				
Poor	274	20 (5.47)	16.705	0.000^*∗*^
Medium	704	15 (2.84)		
Good	68	1 (1.47)		

Age				
1 year	58	5 (6.90)	10.207	0.070
2 years	265	13 (4.15)		
3 years	425	12 (3.29)		
4 years	211	6 (3.32)		
5 years	85	0 (0.00)		
6 years	2	0 (0.00)		

*χ* ^2^ = chi-square, *P* ≥ 0.05 = nonsignificant, and *P* < 0.05 = significant. ^*∗*^indicates differences along the column.

**Table 4:** Mean PCV value of aparasitaemic and parasitaemic animals and parasite species.

Variable	Description	Frequency	Mean PCV ± SE	*t*	*F*	*P* value
Result of BCT	Parasitaemic	36	22.22 ± 0.92	4.462		0.000^*∗*^
	Aparasitaemic	1010	26.18 ± 0.16			

PSANI	*T. vivax*	19	21.526 ± 1.20^a^		9.532	0.000^*∗*^
	*T. congolense*	17	23.412 ± 1.27^ab^			
	Noninfected	1010	26.179 ± 0.17^b^			

^a,b^The values across the column with different superscripts are significantly different from each other (*P* < 0.05). BCT = buffy coat technique and PSANI = parasite species and noninfected. ^*∗*^indicates differences along the column.

**Table 5:** Apparent density of flies caught during the study period.

Study sites	No. of PA's	Altitude range	No. traps	No. days	*Glossina* species					
					*G. morsitans*		*G. tachinoides*		Total	*F*/*T*/*D*
					Male	Female	Male	Female		
Chewaka	6	1129–1355	60	2	81	124	52	70	327	2.73
Meko	3	1420–1593	40	2	6	20	0	0	26	0.33
Dabo Hana	3	1268–1506	60	2	27	67	410	894	1398	11.65
Total	12	—	160	6	114	211	462	964	1751	1.82

*F*/*T*/*D* = fly/trap/day

## Data Availability

The data used to support the findings of this study are included within the article.

## References

[1] Abayneh A. A. (2018). Study on prevalence of bovine trypanosomosis and tsetse density in Kindo Koysha Woreda of Wolaita Zone, Ethiopia. *International Journal of Research Studies in Biosciences (IJRSB)*.

[2] Tesfaheywet Z., Abraham Z. (2012). Prevalence of bovine trypanosomosis in selected district of Arbaminch, SNNPR, Ethiopia. *Global Veterinaria*.

[3] Kumar H., Gupta M. P., Sidhu P. K. (2012). An outbreak of acute *Trypanosoma evansi* infection in crossbred cattle in Punjab, India. *Journal of Applied Animal Research*.

[4] Mulaw S., Mekonnen A., Abebe F. (2011). Study on the prevalence of major trypanosomes affecting bovine in tsetse infested Asosa district of Benishangul Gumuz regional state, western Ethiopia. *Global Veterinaria*.

[5] Abebe G., Jobre Y. (1996). Trypanosomiasis: a treat to cattle production in Ethiopia. *Revue de Médecine Vétérinaire*.

[6] National Tsetse and Trypanosomiasis Investigation and Control Centre, NTTICC (2004). *Annual Report on Tsetse and Trypanosomosis Survey, Bedelle, Ethiopia*.

[7] Meberate A., Menjeta B., Vreysen M., Bencha B., Woldyes G. The distribution and relative abundance of tsetse flies in the Southern Rift valley of Ethiopia: preliminary survey results.

[8] Abebe G. (2005). Trypanosomosis in Ethiopia. *Ethiopian Journal of Biomedical Science*.

[9] STEP, Ministry of Science and Technology (2012). *Southern Tsetse Eradication Project (Step), Field Operation Manual of Tsetse And Trypanosomosis Control and Monitoring, Addis Ababa. Ethiopia*.

[10] Keno M. The current situation of tsetse and trypanosomiasis in Ethiopia.

[11] Tesfaye M. (2002). *Report of Trypanosome Infection Rate in Didessa Valley from Bedele*.

[12] Livestock Development and Marketing Agency (LDMA) (2010). *Annual Progress Report*.

[13] Central Statistical Authority, CSA (2018). *Livestock Population of Ethiopian*.

[14] Pasquini C., Spurgeno T., Pasquini S. (2003). *Anatomy of Domestic Animals: Systematic and Regional Approach*.

[15] Nicholson M., Butterworth M. (1986). *A Guide to Condition Scoring of Zebu Cattle*.

[16] Socioeconomic Data, Dabo Hana District: Annual Report, Kone, 2012

[17] Buno Bedele Zone Livestock and Fisheries Development Office, Annual report, 2018

[18] Chewaka Woreda Agricultural and Rural Development Office, CWARDO, Chewaka Woreda Agricultural and Rural Development Office Report, 2015

[19] Thrusfield M. (2007). *Veterinary Epidemiology*.

[20] Thrustfield M. (2005). *Veterinary Epidemiology*.

[21] Murray M., Murray P. K., McIntyre W. I. M. (1977). An improved parasitological technique for the diagnosis of African trypanosomiasis. *Transactions of the Royal Society of Tropical Medicine and Hygiene*.

[22] Paris J., Murray M., Mcodimba F. (1982). A comparative evaluation of the parasitological technique currently available for the diagnosis of African trypanosomosis in cattle. *Acta Tropica*.

[23] Woo P. T. (1970). The haematocrit centrifuge technique for the diagnosis of African trypanosomiasis. *Acta Tropica*.

[24] Van den Bossche P., Shumba W., Makhambera P. (2000). The distribution and epidemiology of bovine trypanosomosis in Malawi. *Veterinary Parasitology*.

[25] Leak S. G. A. (1999). *Tsetse Biology and Ecology Their Role in the Epidemiology of Trypanosomosis*.

[26] Leak S. G. A., Woume K. A., Colardeue C. (1987). *Determination of Tsetse Challenge and its Relationship with Trypanosomosis Prevalence in Livestock Production in Tsetse Infested Areas of Africa*.

[27] Adane M., Gezahagne M. (2007). Bovine trypanosomosis in three districts of east gojjam zone bordering the blue nile river in Ethiopia. *Journal of Infection in Developing Countries*.

[28] Abebayehu T., Biniam T. (2010). Bovine trypanosomosis and its vectors in two districts of Bench Maji zone, southwestern Ethiopia. *Tropical Animal Health and Production*.

[29] Wondewosen T., Dechasa T., Anteneh W. (2012). Prevalence study of bovine trypanosomosis and tsetse density in selected villages of Arbaminch, Ethiopia. *Journal of Veterinary Medicine and Animal Health*.

[30] Marta T., Bedaso K., Gutu K., Eshetu G. (2016). Prevalence of bovine trypanosomosis and its vector apparent density in Chora district of illuababora western Oromia, Ethiopia. *Journal of Veterinary Medicine and Animal Health*.

[31] Eskziaw B., Alebachew T., Amare B., Ayichew T., Abebaw G. (2016). A study on cattle trypanosomosis and its vectors in Gimbo and Guraferda districts of Southwest Ethiopia. *Journal of Veterinary Medicine and Animal Health*.

[32] Melkamu M., Sisay A., Jelalu K., Yimer M., Ashebr A. (2017). Vector identification and bovine trypanosomosis in Edja district, south Ethiopia. *Livestock Research for Rural Development*.

[33] Megersa L., Feyisa B., Dereje A., Behablom M. (2019). Prevalence of bovine trypanosomosis and apparent density of tsetse fly in botor Tolay district, Jimma zone, Ethiopia. *Biomedical Journal of Scientific & Technical Research*.

[34] Tewodros F., Mitiku T., Tadegegn M., Mersha C. (2012). Prevalence of bovine trypanosomosis and distribution of vectors in Hawa Gelan district, Oromia region, Ethiopia. *Global Veterinaria*.

[35] Zemedkun G., Ayichew T., Alebachew T. (2016). Study on prevalence of bovine trypanosomosis and density of its vectors in three selected districts of Wolaita Zone, southern Ethiopia. *Journal of Veterinary Medicine and Animal Health*.

[36] Urquhart G. M., Armour J., Duncan J. l., Dunn A. M., Jennings F. W. (1996). *Veterinary Parasitology*.

[37] Stephen L. E. (1986). *A Veterinary Perspective*.

[38] FAO (2002). *Food, Agriculture and Food Security, the Global Dimension*.

[39] Van den Bossche P., Rowlands G. J. (2001). The relationship between the parasitological prevalence of trypanosomal infections in cattle and herd average packed cell volume. *Acta Tropica*.

[40] Whitelaw D. D., Gardiner P. R., Murray M. (1988). Extravascular foci of *Trypanosoma vivax* in goats: the central nervous system and aqueous humor of the eye as potential sources of relapse infections after chemotherapy. *Parasitology*.

[41] Hoare C. A. (1972). The trypanosomosis of mammals: a zoological monograph. *International Journal of Research Studies in Biosciences (IJRSB)*.

[42] Food and Agriculture Organization of the United Nation, FAO (2015). Tsetse and trypanosomosis information. *PAAT Programme Against African Trypanosomosis*.

[43] Ayele T., Ephrem D., Elias K., Tamiru B., Gizaw D. (2010). Prevalence of bovine trypanosomosis and its vector density in Daramallo district, southwestern Ethiopia. *Journal of Veterinary Advances*.

[44] Wondewosen T., Dechasa T., Anteneh W. (2012). Prevalence study of bovine trypanosomosis and tsetse density in selected villages of Arbaminch, Ethiopia. *Journal of Veterinary Medicine and Animal Health*.

